# Enhancement of Bond Performance of Advanced Composite Materials Used in Cable Bridge Structures Based on Tensile Tests

**DOI:** 10.3390/ma15082948

**Published:** 2022-04-18

**Authors:** Tae-Kyun Kim, Woo-Tai Jung

**Affiliations:** Department of Structural Engineering Research, Korea Institute of Civil Engineering and Building Technology, 283, Goyang-daero, Ilsanseo-gu, Goyang 10223, Gyeonggi-do, Korea; woody@kict.re.kr

**Keywords:** composite materials, fiber-reinforced polymer (FRP), carbon FRP cable bridge, bond performance, tensile strength, prestressed concrete

## Abstract

Structural steel and concrete are essential materials for the construction of social infrastructures. However, these materials undergo degradation over time, thereby causing steel corrosion. To address this problem, a fiber-reinforced polymer (FRP) is used for reinforcement. In this study, tensile tests were performed to evaluate the material properties for the application of the FRP to cable bridge structures. These tests aimed to investigate various parameters to improve bond performance. Based on experiments with different parameters, sufficient bond performance could be achieved if the following conditions are met: mortar water ≤16%, regardless of the manufacturer; a depth of splitting and steel pipe length ratio ≥75%; upward/downward directions for the mortar injection; and the use of fiber-sheet reinforcement. In addition, the steel pipe used in the test (length of 410 mm and outer diameter of 42.7 mm) performed the best in terms of workability and cost effectiveness. By conducting more accurate tests to study the basic properties of materials, more accurate conditions to accomplish sufficient bond performance can likely be achieved. This will contribute to improved cost effectiveness and safety in the use of carbon FRP cables in cable bridge constructions.

## 1. Introduction

Structural steel and concrete are indispensable materials for the construction of social infrastructures. However, these materials degrade over time [1,2]. Typical degradation phenomena include chloride attacks, carbonation, and freeze–thaw damages [3,4,5]. Chloride attacks and carbonation penetrate the surfaces of aging concrete and induce crack penetration, resulting in structural steel corrosion and degradation of the structural performance [3,4,5]. Freeze and thaw damage can also cause steel corrosion owing to the diffusion of water in the concrete’s cracks and pores. These types of degradation also reduce the service lifetimes of structures drastically [3,4,5]. Further, when steel cables are exposed to the external environment, to which actual bridge structures are exposed, they are highly susceptible to corrosion [6,7]. Therefore, fiber-reinforced polymer (FRP), a noncorrosive material, is applied to resolve this corrosion problem of structural steel. However, although materials such as FRP bars are applied to the construction of real-world structures, they do not have a considerable impact compared with structural steel [8,9].

Research on FRP is ongoing. When FRPs are applied to the cables of cable-supported bridges, tests on the material properties should be conducted using various test methods [8,9], which are as follows: According to the American Concrete Institute (ACI) 440.6-08 (Guide Test Methods for FRPs for Reinforcing or Strengthening Concrete Structures) and the American Association (AASHTO) (Guide Specifications for the Design of Concrete Bridge Beams Prestressed with Carbon Fiber-Reinforced Polymer (CFRP) systems), at least 25 and 50 samples, respectively, are required for tests [10,11]. These samples should be randomly chosen from five manufacturing sites. If there are no major changes, the validity of the test results can last for 3 years [10,11]. The load-resistance tests include a tensile test (for the derivation of strength, elastic modulus, and strain at break), shear test (for the derivation of the transverse shear strength), fatigue test (to investigate fatigue behavior according to the applied stress, number of load cycles, and speed), and interfacial bond behavior test [12,13,14]. Tests conducted to investigate the time-dependent deformation characteristics of materials include creep and stress relaxation tests [12,13,14]. Although there are various tests for testing material properties of FRPs, the tensile strength is the most important property for material applications to bridge cables [15,16,17,18,19]. This is because the tensile strength of the material has the greatest impact on prestressed concrete [20,21,22,23,24,25,26,27].

Existing works related to the application of FRP to cables are as follows. You et al. [28] used glass-fiber reinforced polymer (GFRP) rebar as an alternative material to address the problem of corrosion, conducted a comparative analysis based on tensile tests for various parameters, and performed a validation of the results. Oh et al. [29] experimentally studied fatigue and flexural bonding characteristics of a concrete beam reinforced with GRFP rebars. Rudenko et al. [30] performed nanomodifications of building reinforcing bars of various types to increase the low modulus of elasticity of FRP. Based on the results, they performed comparative analyses based on tensile strength tests. Kim et al. [31] evaluated and comparatively analyzed bond performance with various composite materials, such as glass and basalt, to strengthen concrete structures. Ali et al. analyzed the behavior of the concrete columns subject to the effect of the reinforcement (steel vs. GFRP), the pitch of the GFRP helices, and the addition of nonmetallic fibers in the form of glass and polypropylene fibers [28,29,30,31,32].

International research teams typically perform tests according to the ACI 440.3R-12 (Guide Test Methods for Fiber-Reinforced Polymer (FRP) Composites for Reinforcing or Strengthening Concrete and Masonry Structures) and the American Society for Testing and Materials (ASTM) D7205 (Standard Test Method for Tensile Properties of Fiber Reinforced Polymer Matrix Composite Bars) standards. For the study cases in Korea, FRP tensile tests were performed according to the KS F international standardization organization (ISO) 10406-1 standard (Fiber-reinforced Polymer (FRP) Reinforcement of Concrete—Test Methods—Part 1: FRP Bars and Grids) [12,13,14]. In addition, most of the studies mainly investigated GFRP, although some of them included CFRP as one of the parameters. For most of the test standards, there were detailed descriptions of GRFP, which indicated that for other fiber materials, tests were performed in a similar manner [12,13,14]. Further, among most of the FRP composite material applications to structures, there were cases in which tests on the material properties were not performed, and the property values provided by manufacturers were used instead. However, different types of fibers exhibit different characteristics and properties; therefore, it is imperative to derive an optimal test method for each type of FRP material.

Therefore, in this study, CFRP cables that can be used in bridges were examined based on the considerations of different types of FRPs used in various types of constructions. In the case of CFRP cables, the physical properties of the material must be tested and verified before application to actual structures because the properties may differ according to the manufacturer’s construction methods and environmental conditions. To improve the bond performance of a specimen, its physical properties are tested by varying a diverse range of parameters, such as the mortar manufacturer, depth of splitting against the steel pipe length, and the upward/downward directions of mortar injections and reinforcements of the fiber sheet at the end. Furthermore, to enhance workability based on optimal specimen fabrication parameters, additional performance tests were conducted by varying the dimensions of the steel pipes. Based on more detailed and accurate tests of basic material properties, we expected an accurate estimation of composition to achieve improved bond performance. This will improve the cost effectiveness and safety associated with the use of CFRP in cable bridge constructions. Finally, in this study, a test was conducted using Ø10 circular, single CFRP. However, actual bridge cables are used in various cross-sectional areas and shapes (circular, strand, and others). Therefore, this study can be used to provide basic research data.

## 2. Materials and Methods

### 2.1. FRP

FRP, a composite material, is made of a polymer matrix reinforced with fibers. The most commonly applied fibers contain carbon, glass, and aramid [33,34]. In addition, vinyl ester, polyester, and epoxy resins are used for molding fibers, and for the base material [33,34]. FRPs applied in the construction industry can be classified into CFRP, GFRP, and aramid fiber-reinforced polymer according to the fibers used. The mechanical/physical properties of each type of FRP are outlined in ACI 440R-96 [35]. When these composite materials are used in the construction industry, they are processed into various shapes, such as sheets, plates, reinforcing bars, and tendons. Among the different types of FRPs, the CFRP used in place of structural steel in this study exhibits light weight, high-fatigue resistance, low-maintenance cost, high-tensile strength, low-thermal expansion, corrosion resistance, and high-durability characteristics [33,34]. Additionally, in this study, a test was conducted using Ø10 circular, single CFRP. The fiber was carbon, the resin was epoxy, the fiber volume ratio was approximately 65%, and the maximum strain was approximately 1.5%.

### 2.2. Specimen Preparation Process

**Figure 1:**
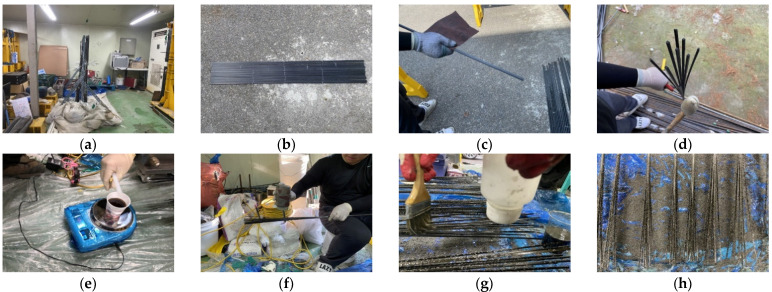

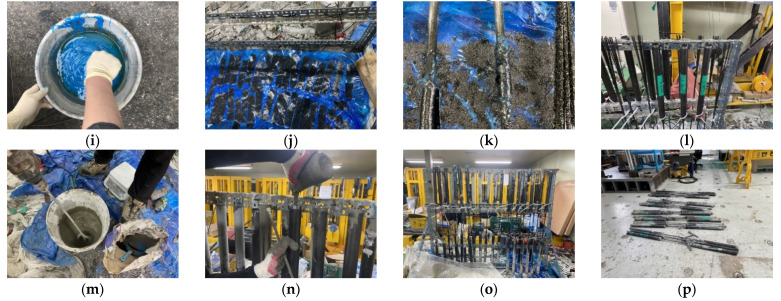
Preparation process for carbon fiber-reinforced polymer (CFRP) specimens used for tensile tests: (**a**) Assembly of holder, (**b**) CFRP cutting, and (**c**) CFRP interface treatment. (**d**) Division of CFRP specimen into nine equal parts. (**e**) Epovia resin mixer, (**f**) first oxide costing, (**g**) second oxide costing, (**h**) completion of oxide coating, (**i**) EPONDEX resin mixing, (**j**) resin coating of the FRP sheet, (**k**) completion of the FRP sheet attachment, (**l**) mounting of the specimen, (**m**) mixing of mortar, (**n**) specimen placement, (**o**) state after placement, and (**p**) completion of the test specimen preparation process.

Figure 1 summarizes the process used to prepare CFRP round steel pipe specimens for tensile tests. The specimen preparation process involved the following steps. A holder was installed to fix the specimen, the interface of the CFRP specimen attachment was sanded, and the CFRP specimen was divided into nine equal parts to increase the area of the CFRP attachment cross-section. Subsequently, an Epovia resin mixer was prepared, and the first Epovia resin and oxide coating were applied. This was followed by the application of the second Epovia resin and oxide coating, the curing of the Epovia resin, mixing of the EPONDEX resin, application of the EPONDEX resin on the FRP sheet and CFRP bar-end finish, curing the EPONDEX resin, and the assembling and mounting of the CFRP and steel pipe mold and mortar mixer; in turn, these were placed on one side of the steel pipe mold followed by curing, placement on the other side of the steel pipe mold and curing, and detachment from the holder after the completion of the curing process.

### 2.3. Tensile Test Method with the Sample Specimens

**Figure 2:**
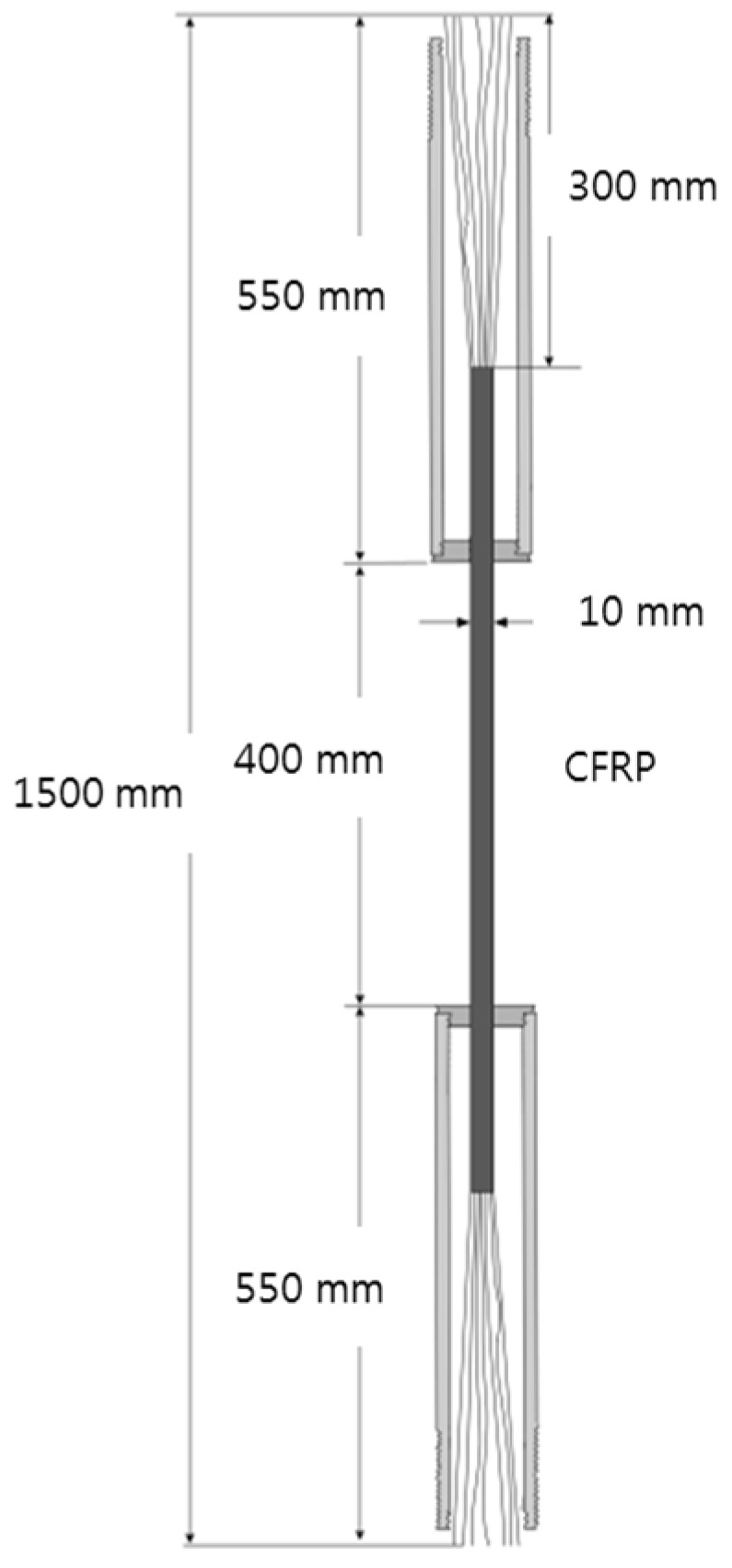
Dimensions of a typical sample specimen.

Figure 2 shows a sample specimen of the CFRP round steel pipe. The prepared specimen had a total length of 1500 mm, and the steel pipe had a length of 550 mm. The lengths of the nine equally divided CFRP parts were equal to 300 mm, and the lengths of the top and bottom ends in the center were equal to 400 mm.

**Figure 3:**
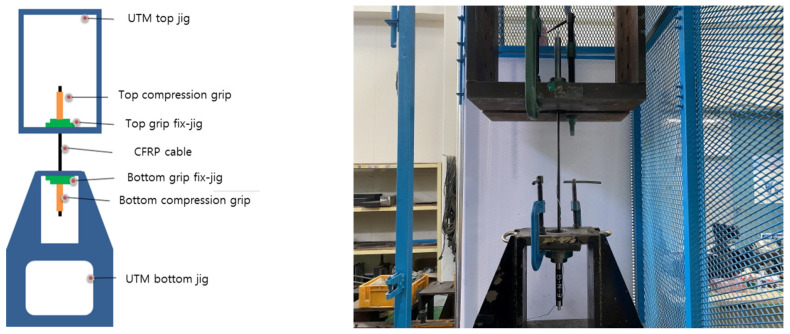
Tensile test setup for round steel pipe mold specimens [36].

Figure 3 shows a schematic of the test setup for the specimens of the round steel pipe mold. For the measurement of the specimens, the static electric resistance type device TDS-530 (TDS-530 Data Logger, Tokyo Sokki Kenkyujo, Tokyo, Japan), which is the most commonly used equipment for real-world measurements of structures, was used. In the center of the CFRP cable, a strain gauge (TML Strain Gauge, Tokyo Sokki Kenkyujo, Tokyo, Japan) was attached. The main dimensions of the strain sensor were as follows: the base size was 10 mm in length and 3 mm in width, and the size of the thin plate used for strain sensing was 5 mm in length and 1.5 mm in width. The resistance value was 120. The specimen was fixed at the top and bottom jigs. After mounting the CFRP cable, the specimen was fixed using grips at the compression points at the top and bottom parts of the device. The fixed round steel pipe mold was tested using a 1000 kN Universal Testing Machine (UTM). Displacement control was performed at the loading rate of 5 mm/min. In addition, the test was conducted until we decided to terminate it.

### 2.4. Tensile Test Results with the Sample Specimen

**Figure 4:**
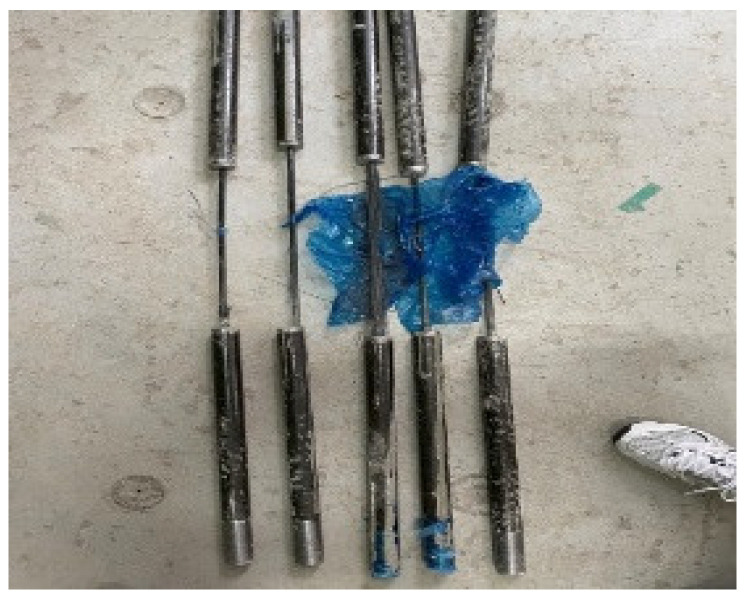
Fracture location image.

**Figure 5:**
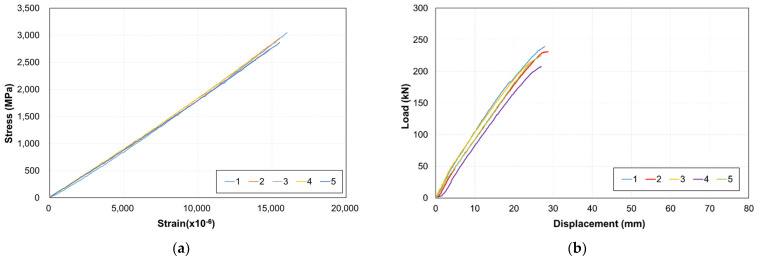
Sample specimen tensile test results: (**a**) stress and strain and (**b**) load–displacement curves.

Figure 4 shows a photograph of fractured samples and the fracture locations. Table 1 and Figure 5 present the tensile test results of the sample specimens. Five specimens were tested after curing for 7 days. The average values of the maximum load, tensile strength, and modulus of the specimens were 224 kN, 2851 MPa, and 187 GPa, respectively. The ratios of the maximum and minimum values of each outcome were 87%, 87%, and 97%. The maximum load values exhibited some deviation. Furthermore, the fractures occurred at the end of the specimen rather than at the center, thus indicating the occurrence of slip. However, the load–displacement curves of different specimens yielded similar shapes. This can be attributed to the insufficient CFRP bond strength and to the insufficient curing period of the mortar. Therefore, in this experiment, we aimed to improve the bond performance by setting various parameters.

## 3. Experiments for the Improvement of Cable Bond Performance

### 3.1. Variation of Parameter Values with Respect to the Fabrication Method of the CFRP Bond Performance Specimens

**Figure 6:**
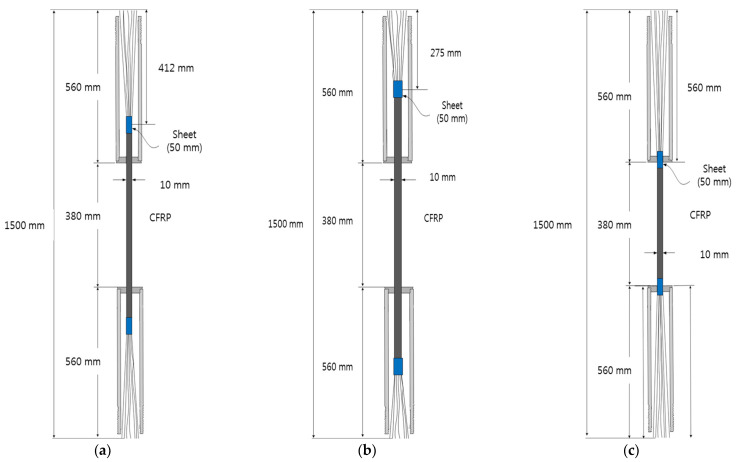
Schematics of sample specimens with respect to the depth of specimen splitting: Cases (**a**) 1, (**b**) 2, and (**c**) 3.

Table 2 shows the parameters for cable. Specimens were prepared for seven different cases, and three specimens were fabricated in each case. For the mortar types, manufacturers A and B were selected. For manufacturer A, the ratio of water used in the mortar was 14%, and for manufacturer B, the corresponding ratios were 16% and 18%. The ratios of the depth of splitting against the steel pipe length were equal to 50%, 75%, and 100%. The directions of mortar injection were classified as upward and downward. Finally, the presence/absence of the fiber sheet reinforcement was also set as another parameter. Figure 6 shows the dimensions of the reinforced specimen based on the sample specimen. The total length was 1500 mm, the steel pipe length was 560 mm, and the length between the top and bottom ends in the center was 380 mm, which was the same for all cases.

**Figure 7:**
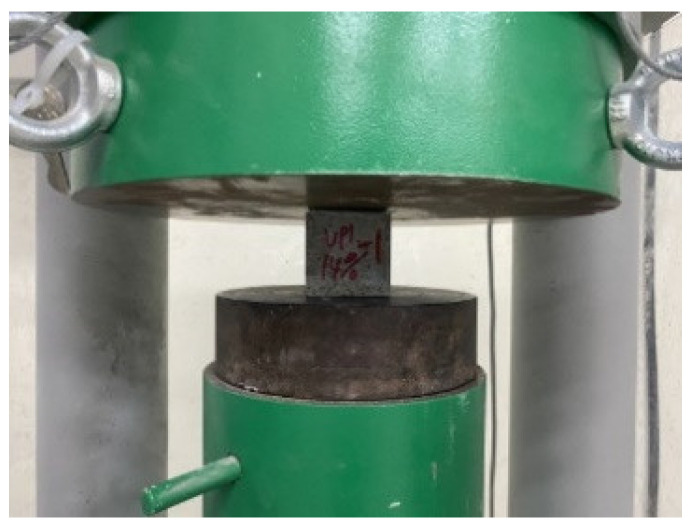
Cubic mold compressive strength test.

**Figure 8:**
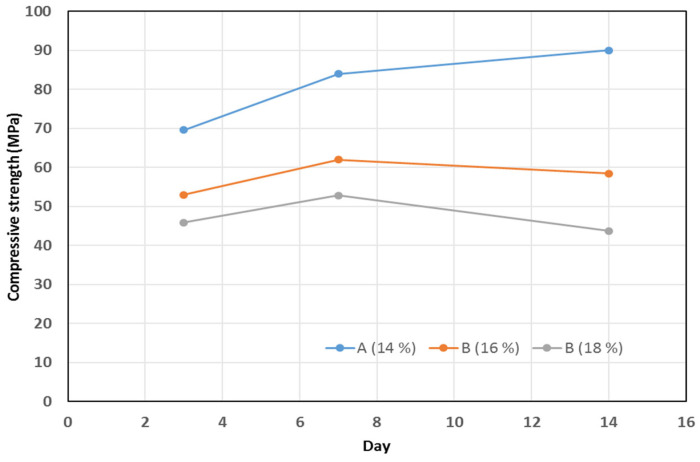
Compressive strength plots of the cubic mold specimens.

Figure 7 shows the strength test of mortar for manufacturers A, B (16 %), and B (18 %). The test used a 300 ton compression UTM. Table 3 presents the compressive strength values for mortar at 3, 7, and 14 days. The method of the compressive strength test was in accordance with KS F 4042 (Polymer Cement Mortar Compressive Strength Test for Repairing Concrete Structures) [37]. Figure 8 is a graphical representation of the compressive strength. The average 3-day strengths for products A (14%), B (16%), and B (18%) were 69.6, 53.0, and 45.9 MPa, respectively; thus, the strength of product A (14%) was approximately 1.5 times higher than that of product B (18%). The average 7-day strengths for the products A (14%), B (16%), and B (18%) were 84.0, 62.0, and 52.8 MPa, respectively; thus, the strength of product A (14%) was approximately 1.6 times higher than that of product B (18%). The average 14-day strength for products A (14%), B (16%), and B (18%) were 90.0, 58.4, and 43.7 MPa, respectively; thus, the strength of product A (14%) was approximately two times higher than that of product B (18%). In the case of the 14-day strength, product B (16%) and B (18%) exhibited decreases in their strengths by 0.94 and 0.83 times, respectively, compared with their 7-day strengths. This can be attributed to the use of only three specimens in the tests. Therefore, it is expected that more accurate results can be derived if the tests are conducted using more specimens. In addition, two test specimens, A (14%) and B (18%), were used because they were wrong when the mold was deformed. In addition, the results of the compression strength test of the product from manufacturer A were excellent. Furthermore, low-water content is more advantageous in terms of strength development.

### 3.2. Test Results with Respect to Different Parameters for Assessing CFRP Bond Performance

Table 4 shows the bond performance results of the tested specimens. The test results include the maximum load, tensile strength, modulus of elasticity, and fracture type. The details of the control specimen (Case 1) are as follows: mortar from manufacturer A, depth of splitting of 75%, upward direction of mortar injection, and fiber-sheet reinforcement. The maximum load in the Case 1 specimen was 268.6 kN. The details of the specimen of Case 3 are as follows: mortar from manufacturer A, depth of splitting of 100%, upward direction of mortar injection, and fiber-sheet reinforcement. The maximum load in the Case 3 specimen was 265.9 kN, which is equal to 0.99 times that of the control specimen. The details of the specimen in Case 4 are as follows: mortar from manufacturer A, depth of splitting of 75%, downward direction of mortar injection, and fiber-sheet reinforcement. The maximum load of the specimen in Case 4 was 269.5 kN, which is 1.01 times that of the control specimen. The details of the specimen in Case 6 are as follows: mortar from manufacturer B (16%), depth of splitting of 75%, upward direction of mortar injection, and fiber-sheet reinforcement. The maximum load of the specimen of Case 6 was 268.4 kN, which is 0.99 times that of the control specimen. As it can be observed from these values, Cases 3, 4, and 6 exhibit almost the same maximum load as the control specimen. However, in Case 2 (mortar from manufacturer A, depth of splitting of 50%, upward direction of mortar injection, and fiber-sheet reinforcement), the maximum load was 265.9 kN, which is 0.62 times that of the control specimen. In Case 5 (mortar from manufacturer A, depth of splitting of 75%, upward direction of mortar injection, without fiber-sheet reinforcement), the maximum load was 254.2 kN, which is 0.94 times that of the control specimen. Finally, in Case 7 (mortar from manufacturer B (18%), depth of splitting of 75%, upward direction of mortar injection, and with fiber-sheet reinforcement), the maximum load was 266.2 kN, which is 0.99 times that of the control specimen.

Therefore, upon examination of Cases 3, 4, and 6, it can be observed that the following conditions should be satisfied for the specimen to exhibit sufficient tensile load: for mortar, the amount of water used should be no more than 16% regardless of the manufacturer, the depth of splitting should be not less than 75%, and the direction of mortar could be either upward/downward. Finally, the condition with the fiber-sheet reinforcement should be applied. As in Case 2, when the depth of splitting became 50% (less than 75%), the maximum load decreased rapidly. As in Case 5, without the fiber-sheet reinforcement at the end part, the maximum loads of these specimens were smaller than those of the control specimen. Furthermore, in Case 7, the average maximum load was similar to that of the control specimen. However, examination of the specimens showed that there was a considerable decrease in strength. Accordingly, it was considered that additional tests are required for further investigation of the differences.

Tensile strength was obtained by dividing the maximum load by the cable’s cross-sectional area. Given that the same specimen was used for all cases, the trend in the tensile strength was the same as the maximum load. The strength values of Cases 3, 4, and 6 were higher, but those of Cases 2 and 5 were smaller than the corresponding values of the control specimen. In terms of the modulus of elasticity, the values were similar or higher than the corresponding values of the control specimen for most cases, but for the specimen in Case 2, the value of modulus of elasticity was reduced by approximately 5%.

Regarding the type of fracture, the control specimen and most other specimens exhibited flower-shaped fractures in their center parts. This confirms that an equal load was applied to the top and bottom steel pipe molds during the tensile test. However, in Case 2, as the depth of splitting was reduced to 50%, slip occurred in which the CFRP fell off rather than fractured owing to the decrease in the specimen’s bond strength.

**Figure 9:**
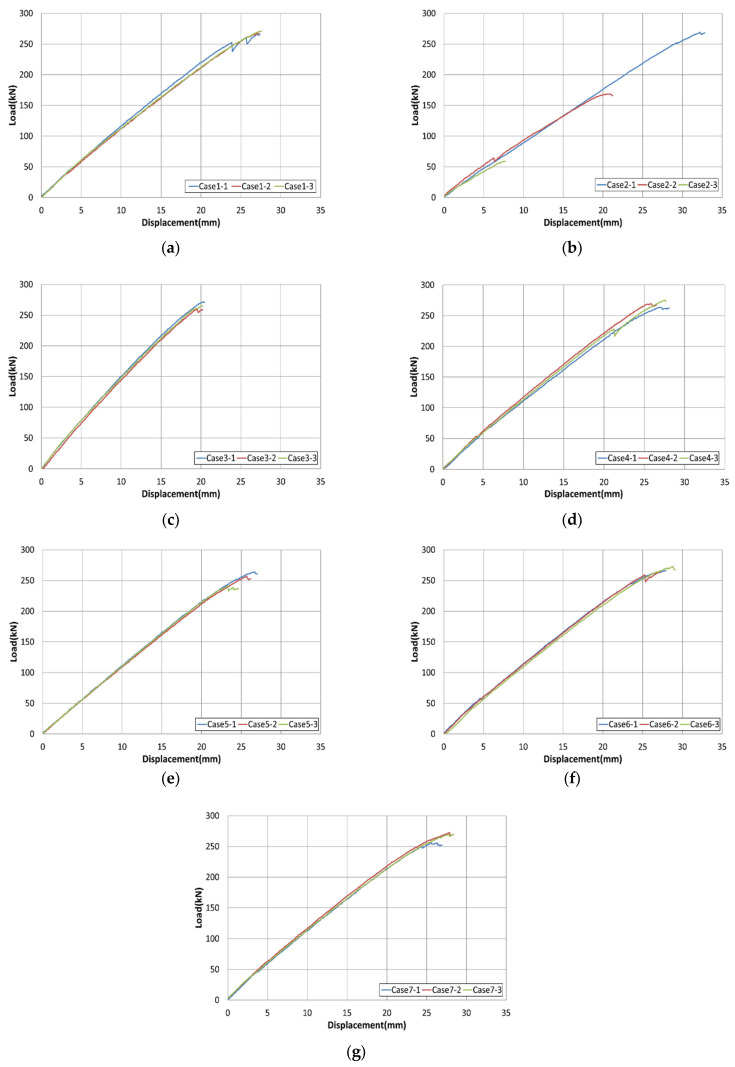
Load–displacement graphs used for the assessment of bond performance improvements: Cases (**a**) 1, (**b**) 2, (**c**) 3, (**d**) 4, (**e**) 5, (**f**) 6, and (**g**) 7.

Figure 9 shows the load–displacement curves used for the assessment of bond performance improvement. In the case of the control specimen and Cases 4–7, the maximum displacements were 27.3 (C), 26.8, 25.2, 28.0, and 27.3 mm, respectively. Therefore, only the maximum load values were different in these cases; by contrast, the values of stiffness (derived from the slope of the curve) until failure were similar. However, in Case 3, the displacement was 20.1 mm, which was 0.73 times smaller than that of the control specimen, but the value of stiffness until failure was considerably higher. Conversely, Case 2 shows an average displacement of 20.0 mm, which is similar to Case 3, but in the case of each specimen, the displacement values were 32.2, 20.7, and 7.4 mm; as indicated, a considerable variation exists among the values due to slipping. Therefore, based on Table 4 and Figure 9, for improved bond performance and optimal workability, the required conditions are as follows: for mortar, the amount of water used should be no more than 16% regardless of the manufacturer, the depth of splitting should be not less than 75%, the direction of mortar could be either upward/downward, and fiber-sheet reinforcement ought to be used. In addition, it was considered that bond performance would also be affected by the dimensions of round steel pipe mold, and additional tests with different parameters were conducted based on the optimal construction method.

### 3.3. Experiments with Different CFRP Round Mold Dimensions

**Figure 10:**
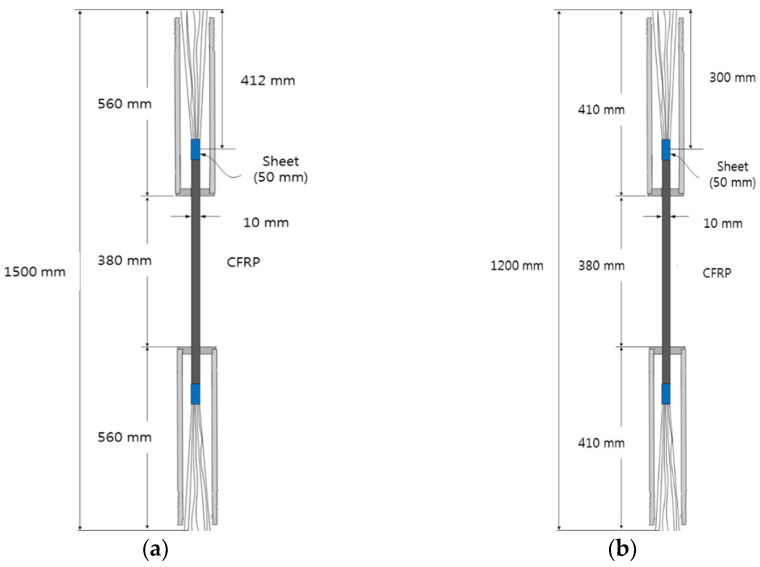
Specimen dimensions with respect to the steel pipe parameters: (**a**) Cases A–C, and (**b**) Case D.

**Figure 11:**
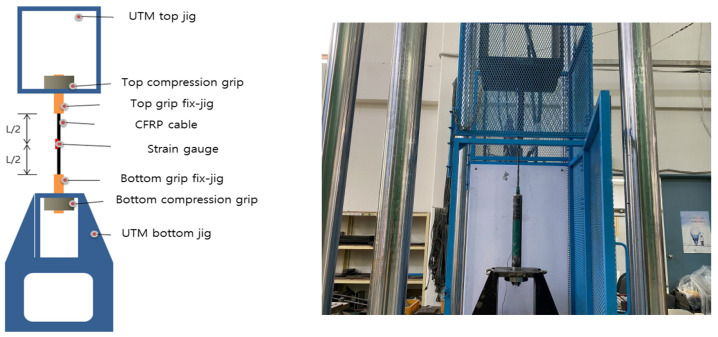
Setup of steel pipe tensile test.

As shown in Table 5, four types of parameters were selected according to the dimensions of KS D 3562 (Carbon Steel Pipes for Pressure Service) for the CFRP round steel pipe mold [38]. In Case A, the specimens were prepared in the same way as the optimal specimen preparation method. Moreover, in Case B, the thickness was set to 6.4 mm, which was different than that in Case A. Case C set the outer diameter at 60.5 mm and the thickness at 8.7 mm. In the last case, Case D had a steel pipe length of 410 mm, an outer diameter of 42.7 mm, and a thickness of 4.9 mm. In addition, all four specimens were fabricated with a screw tab pitch of 1.5 mm and a screw tab length of 100 mm. Figure 10 shows the dimensions of the specimen. Figure 11 shows the experimental setup for the tensile test. In the experiment conducted previously wherein the specimen parameters were varied, the neck part of the steel pipe mold was fixed to conduct the tensile tests. However, for the experiments of Cases A to C with respect to the round mold dimensions, the tensile tests were performed by fixing the screw tabs as shown in Figure 11. However, in Case D, the neck part was fixed, as shown in Figure 3 (and was the same as the previous method), to conduct the tensile test. Three specimens were prepared in each case.

### 3.4. Experimental Results with Different CFRP Round Mold Dimensions

Table 6 presents the experimental results with respect to the round mold dimensions. The average maximum load, tensile strength, and elastic modulus of the specimen in Case A were 192.8 kN, 2456.1 MPa, and 179.9 GPa, respectively. Furthermore, the maximum load values for Cases B to D were 281.7, 278.1, and 275.0 kN, respectively, which were 1.46, 1.44, and 1.43 times higher than those of Case A. Given that tensile strength was also derived based on the maximum load value, it exhibited a similar behavior to that of maximum load; thus, the values were 1.46, 1.44, and 1.43 times higher than those of Case A. The modulus of elasticity values were 181.9, 183.9, and 182.8 GPa, which were approximately 1.01, 1.02, and 1.02 times higher than those of Case A, respectively. Therefore, in Cases 2 and 3, as the outer diameter and thickness of the steel pipe increased, the performance increased. In addition, as in Case D, even when the length of the steel pipe was reduced compared with Case A, it was observed that, depending on the part to be fixed during the tensile test, there was a difference in the values of the maximum tensile load.

As for the type of fracture, with Case A dimensions, when the screw tab was used, a tensile test could not be performed properly as the steel pipe developed fractured in the middle of the test. In Case B, flower-shaped fracture occurred. Case C also had flower-shaped fracture (slip). Case D had flower-shaped fracture. Furthermore, in the manufacturing process (workability) of specimen preparation and placement, as the outer diameter and thickness of the steel pipe increased, the weight increased and caused inconvenience in its use. In Case D, the workability was considerably better as the length of the steel pipe decreased. Additionally, compared with Cases 1 to 7, outlined in Table 4 according to the parameters of the bond performance specimens, the maximum load and tensile strength values were approximately 1.02, 1.66, 1.03, 1.02, 1.08, 1.02, and 1.03 times higher, respectively, and the modulus of elasticity values were approximately 1.00, 1.00, 1.00, 0.98, 1.01, 1.00, and 1.00 times higher, respectively, indicating almost similar values. Therefore, Case D is the most suitable condition for the tensile strength test.

**Figure 12:**
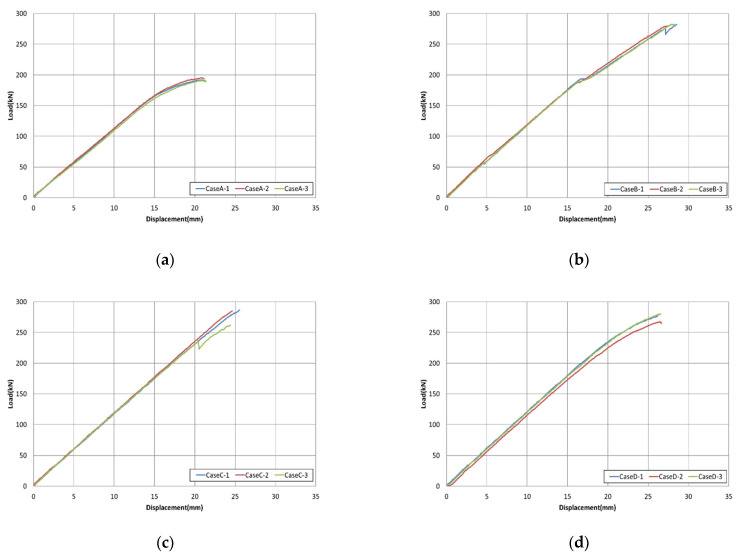
Load–displacement curves with the round mold steel pipe for bond performance improvements: Cases (**a**) A, (**b**) B, (**c**) C, and (**d**) D.

Figure 12 presents the load–displacement curves for different round steel pipe mold parameters. In Case A, the test was terminated at a maximum load of 192.8 kN and a displacement of 20.9 mm owing to steel pipe failure. Cases B and D show similar load–displacement curves, and the values of the load and displacement were 281.7 and 275.0 kN and 27.9 and 26.4 mm, respectively, and yielded similar slope stiffnesses. The values of load and displacement of Case C were 278.1 kN and 24.9 mm, thus indicating smaller displacements compared with Cases B and D, but the slope stiffness was higher. However, it was considered that this case was not suitable as a specimen, owing to workability problems.

## 4. Conclusions

In this study, various CFRP cable tests were performed to gauge the improvement in bond performance with different methods. Experiments were conducted by varying parameters, such as the fabrication method and dimensions of the steel pipe mold. Based on the experiments, the following results were derived.

(1) When the compressive strengths of mortars from manufacturers, A (14%), B (16%), and C (18%) were compared, the average 3-day strength of the product A was 1.5 times that of product C, the average 7-day strength of the product A was 1.6 times that of the product C, and the average 14-day strength of the product A was 2.0 times that product C. Evidently, the strengths of products from manufacturer A were superior, and as the amount of water decreased, the strength response improved.
(2) From the tests performed to compare the bond strength based on different fabrication methods, the following conditions need to be satisfied for sufficient expression of the bond performance: the amount of water used to make mortar should not be more than 16%, regardless of the manufacturer; the depth of splitting should not be less than 75%; the direction of mortar could be either upward or downward; and fiber-sheet reinforcement ought to be used. For the control specimen and most of the other specimens, flower-shaped fracture occured in the center part that indicated application of equal loading at the top and bottom steel pipe mold parts during tensile testing.
(3) Regarding the experiments conducted based on variations of the parameters of round steel pipe mold, steel pipe failure occurred in Case A; in Cases B, C (slip), and D, flower-shaped fractures were observed. Moreover, in the process of specimen preparation and placement, larger dimensions caused the weight of the steel pipe to increase. This caused inconvenience in its use. In Cases B and C, there was considerable inconvenience in the process of preparation and placement. In Case D, workability improved as the length of the steel pipe decreased, and a superior performance was achieved compared with other specimens.
(4) Finally, in this study, various experiments were conducted with different parameters aimed to increase bond performance and the maximum CFRP load. Therefore, in future research, additional studies need to be conducted on more diverse specimen fabrication parameters and methods, and on the dimensions of steel pipe mold, so that they can be applicable to all types of fibers.

## Figures and Tables

**Table 1:** Tensile test results of the sample specimen.

Specimens	CFRP Cable			
	Maximum Load (kN)	Tensile Strength (MPa)	Young’s Modulus (GPa)	Fracture Location
1	239	3048	190	fracture (slip)
2	231	2945	190	fracture (slip)
3	215	2743	184	fracture (slip)
4	208	2654	187	fracture (slip)
5	225	2868	184	fracture (slip)
Avg.	224	2851	187	-

**Table 2:** Setting parameters for cable bond performance improvement.

Case	Type of Mortar			Depth of Splitting			Direction of Mortar Injection		Fiber Sheet Reinforcement	
	(Amount of Water %)			(Ratio to Steel Pipe Length)			Upward	Downward	Y	N
	Manufacturer A	Manufacturer B								
	14%	16%	18%	50%	75%	100%				
Case1 (C)	O				O		O		O	
Case 2	O			O			O		O	
Case 3	O					O	O		O	
Case 4	O				O			O	O	
Case 5	O				O		O			O
Case 6		O			O		O		O	
Case 7			O		O		O		O	

**Table 3:** Results of compressive strength test with cubic mold.

Manufacturers	Day	Compressive Strength (MPa)			
		# 1	# 2	# 3	Avg.
A (14%)	3	66.2	70.6	72.1	69.6
	7	88.3	79.7	84.0	84.0
	14	96.3	93.3	-	90.0
B (16%)	3	50.6	54.0	54.3	53.0
	7	68.3	58.8	58.8	62.0
	14	55.6	57.6	62.0	58.4
B (18%)	3	46.1	44.4	47.3	45.9
	7	55.0	55.0	48.5	52.8
	14	49.1	38.2	-	43.7

**Table 4:** Bond performance improvement outcomes pertaining to the tested specimens.

Specimens	Maximum Load (kN)	Tensile Strength (MPa)	Young’s Modulus (GPa)	Fracture Image
Case 1-1	267.0	3401.3	178.8	flower-shaped fracture
Case 1-2	267.7	3410.2	180.5	flower-shaped fracture
Case 1-3	271.1	3453.5	185.3	flower-shaped fracture
**Case-1 Avg. (Avg.)**	**268.6**	**3421.7**	**181.5**	
Case 2-1	269.0	3426.8	183.3	flower-shaped fracture
Case 2-2	168.6	2147.8	172.4	slip
Case 2-3	59.0	751.6	170.5	slip
**Case-2 Avg.**	**165.5**	**2108.3**	**175.4**	
Case 3-1	271.9	3463.7	182.3	flower-shaped fracture
Case 3-2	259.9	3310.8	179.1	flower-shaped fracture
Case 3-3	265.8	3386.0	185.3	flower-shaped fracture
**Case-3 Avg.**	**265.9**	**3387.3**	**182.2**	
Case 4-1	263.4	3355.4	186.3	flower-shaped fracture
Case 4-2	269.5	3433.1	184.6	flower-shaped fracture
Case 4-3	275.5	3509.6	183.5	flower-shaped fracture
**Case-4 Avg.**	**269.5**	**3433.1**	**184.8**	
Case 5-1	264.1	3364.3	180.7	flower-shaped fracture
Case 5-2	257.2	3276.4	177.4	flower-shaped fracture
Case 5-3	241.4	3075.2	184.9	flower-shaped fracture
**Case-5 Avg.**	**254.2**	**3238.2**	**181.0**	
Case 6-1	266.2	3391.1	185.1	flower-shaped fracture
Case 6-2	266.3	3392.4	178.7	flower-shaped fracture
Case 6-3	272.9	3476.4	184.5	flower-shaped fracture
**Case-6 Avg.**	**268.4**	**3419.1**	**182.8**	
Case 7-1	256.1	3262.4	182.5	flower-shaped fracture
Case 7-2	272.5	3471.3	184.1	flower-shaped fracture
Case 7-3	269.9	3438.2	182.6	flower-shaped fracture
**Case-7 Avg.**	**266.2**	**3391.1**	**183.1**	

**Table 5:** Parameters of the round mold specimen.

Specimens	Case A	Case B	Case C	Case D
steel pipe length (mm)	560	560	560	410
outer diameter (mm)	42.7	42.7	60.5	42.7
thickness (mm)	4.9	6.4	8.7	4.9
screw tab pitch (mm)	1.5	1.5	1.5	1.5
screw tab length (mm)	100	100	100	100

**Table 6:** Bond performance outcomes for tested specimens.

Specimens	Maximum Load (kN)	Tensile Strength (MPa)	Young’s Modulus (GPa)	Fracture Image
Case A-1	192	2445.9	180.1	steel pipe failure
Case A-2	195.1	2485.4	177.1	steel pipe failure
Case A-3	191.3	2426.9	182.5	steel pipe failure
**Case-A Avg.**	**192.8**	**2456.1**	**179.9**	
Case B-1	282.3	3596.2	181.6	flower-shaped fracture
Case B-2	279.8	3564.3	182.3	flower-shaped fracture
Case B-3	283.0	3605.1	181.8	flower-shaped fracture
**Case-B Avg.**	**281.7**	**3588.5**	**181.9**	
Case C-1	286.6	3651.0	186.2	flower-shaped fracture
Case C-2	285.1	3631.8	183.1	fracture (slip)
Case C-3	262.5	3343.9	182.4	flower-shaped fracture (slip)
**Case-C Avg.**	**278.1**	**3542.7**	**183.9**	
Case D-1	277.2	3531.2	182.1	flower-shaped fracture
Case D-2	267.6	3408.9	183.6	fracture (slip)
Case D-3	280.2	3569.4	182.6	flower-shaped fracture
**Case-D Avg.**	**275.0**	**3503.2**	**182.8**	

## Data Availability

The data presented in this study are available upon request from the corresponding author.
